# Changes in Body Condition of Hibernating Bats Support the Thrifty Female Hypothesis and Predict Consequences for Populations with White-Nose Syndrome

**DOI:** 10.1371/journal.pone.0021061

**Published:** 2011-06-22

**Authors:** Kristin A. Jonasson, Craig K. R. Willis

**Affiliations:** Department of Biology, Centre for Forest Interdisciplinary Research (C-FIR), University of Winnipeg, Winnipeg, Canada; Texas A&M University, United States of America

## Abstract

White-nose syndrome (WNS) is a new disease of bats that has devastated populations in eastern North America. Infection with the fungus, *Geomyces destructans*, is thought to increase the time bats spend out of torpor during hibernation, leading to starvation. Little is known about hibernation in healthy, free-ranging bats and more data are needed to help predict consequences of WNS. Trade-offs presumably exist between the energetic benefits and physiological/ecological costs of torpor, leading to the prediction that the relative importance of spring energy reserves should affect an individual's use of torpor and depletion of energy reserves during winter. *Myotis lucifugus* mate during fall and winter but females do not become pregnant until after spring emergence. Thus, female reproductive success depends on spring fat reserves while male reproductive success does not. Consequently, females should be “thrifty” in their use of fat compared to males. We measured body condition index (BCI; mass/forearm length) of 432 *M. lucifugus* in Manitoba, Canada during the winter of 2009/2010. Bats were captured during the fall mating period (n = 200), early hibernation (n = 125), and late hibernation (n = 128). Adult females entered hibernation with greater fat reserves and consumed those reserves more slowly than adult males and young of the year. Consequently, adult females may be more likely than males or young of the year to survive the disruption of energy balance associated with WNS, although surviving females may not have sufficient reserves to support reproduction.

## Introduction

Organisms must balance energy intake against expenditure in order to survive, and available energy must be partitioned between maintenance, somatic growth and reproduction. The balance of energy acquisition and consumption is relatively simple for hibernating endotherms during the winter. Hibernators accumulate a fat store or food cache during the active season and hibernation reduces over-winter energy expenditure to the point where stored energy reserves can balance winter energy costs [1]. Any increase in energy expenditure, however, has the potential to compromise survival [2], [3]. White-nose Syndrome (WNS) is a new disease of bats which appears to cause just such an increase in energy expenditure leading to perhaps the most rapid wildlife population declines ever recorded [4], [5]. In only the first several years of the disease, population declines of 30 to 99% (averaging 73%) have been documented at hibernacula in the northeastern United States [5], [6]. The affliction is associated with a fungal pathogen, *Geomyces destructans*, that invades exposed skin of the face and wing membranes [7], [8]. Although the direct cause of mortality is poorly understood, affected bats are emaciated and have presumably used their hibernation energy stores too rapidly [6]. Preliminary evidence suggests that bats infected with *G. destructans* warm up from torpor more frequently and/or for longer periods during hibernation than unaffected bats, which would explain their more rapid fat depletion [9], [10]. In addition to understanding the proximate causes of mortality, there is a need to generate hypotheses about how WNS may affect populations on a longer timescale. This requires the study of reference populations that have not yet been impacted by the disease [5], [11].

Drastic reductions in body temperature (T_b_) and metabolic rate during torpor can reduce the energy requirements of hibernators to as low as ∼1% of what is needed to defend euthermia [12], [13]. Torpid metabolism accounts for only 10–30% of energy consumption during hibernation while brief arousals to euthermic T_b_ account for the remaining majority of energy expenditure [12]. Prolonged, deep torpor will accrue the greatest energetic savings but also results in physiological/ecological costs. The inability to drink or urinate while torpid results in the buildup of metabolic wastes and dehydration stress [14], [15]. As torpor progresses a sleep debt accumulates [16], [17] and a decline in synaptic contacts [18], [19] may result in partial memory loss [20], but see [21]. Immune function is diminished during torpor [22] and the need to activate it may be one factor that triggers arousals [23], [24]. Responsiveness to sensory stimuli, coordination and motor ability are all reduced, which may increase vulnerability to predation (although see [25], [26]) or rapid environmental changes (e.g., flooding of the hibernaculum) [27].

This trade-off between the energetic benefits and the ecological/physiological costs of torpor has led to the hypothesis that torpor should be expressed at an intermediate rather than maximal level during hibernation if the added energetic costs can be met [28]. A key prediction of this torpor optimization hypothesis is that, when excess energy is available, it should be used to reduce torpor depth and duration, and this is supported by recent experimental studies. For example, free-ranging eastern chipmunks (*Tamias striatus*) with supplemented food caches, exhibited shorter, shallower torpor bouts than controls [29], [30]. Laboratory studies of big brown bats (*Eptesicus fuscus*) and field observations of little brown bats (*Myotis lucifugus*) have also revealed that individuals with more available energy select warmer microclimates that would reduce torpor depth and duration [31]. The torpor optimization hypothesis also predicts that individuals should increase torpor expression during hibernation if spring fat reserves are important for survival and/or reproduction [28]. For example, adult male Richardson's ground squirrels (*Spermophilus richardsonii*) which compete intensely for estrous females in the spring, experience a slower rate of fat depletion than adult females during hibernation [32]. However, despite the accumulating evidence that the expression of torpor during hibernation should and does vary between individuals, deep, prolonged torpor may still be considered the “best” strategy for hibernators during winter [33], [34], [35].

To test Humphries et al.'s (2003) prediction that the need for spring fat reserves should influence energy expenditure during hibernation we examined the effect of age/sex class on the use of energy reserves in an uninfected population of the species which appears most susceptible to WNS, the little brown bat (*Myotis lucifugus*). Little brown bats hibernate in caves or abandoned mines and may migrate up to 220 km from summer roosts to hibernacula [36]. Prior to entrance into hibernation (i.e., immergence) bats deposit additional fat and mate promiscuously, often around hibernacula entrances during a period termed “swarming” [37]. Male and female little brown bats invest their energy differently throughout the year. Although both sexes must acquire sufficient fat reserves to survive the winter, the timing of their reproductive investment differs. Male investment consists of spermatogenesis from May to late August [38], as well as mating during the fall swarming period and, to some extent, with torpid females during hibernation [39]. Sperm is stored in the female reproductive tract throughout hibernation; ovulation and fertilization occur a few days after emergence from hibernation and parturition takes place 50–60 days later [40], [41], [42], [43]. Kunz, Wrazen & Burnett [37] argued that females must retain adequate fat upon emergence from hibernation to mediate the hormonal changes necessary to stimulate ovulation. Female little brown bats cannot adjust their investment by decreasing litter size like other mammals [44] because they have a single pup annually [45], although there is some evidence of embryo resorption and abortion [46], [47]. Therefore, female reproduction is essentially an all-or-nothing event. This should create strong selective pressure for females to be more conservative with their fat reserves during winter, especially given that winter length can vary and females may require an energetic buffer to support reproduction following longer winters. Males, on the other hand, are able to invest in reproduction earlier during hibernation and more incrementally because they can adjust mating effort to match energy availability. This leads to what we term “the thrifty female hypothesis” that adult females should maximize energy savings and rely more heavily on deep torpor during hibernation, while adult males should spend more energy to avoid physiological/ecological costs of torpor and therefore use their fat reserves more quickly during winter.

The torpor optimization hypothesis also predicts differences in hibernation patterns of young-of-the-year (YOY) compared to adults. For little brown bats, YOY of both sexes enter hibernation with proportionally less fat than adults [37], [48], [49] and experience greater over-winter mortality [50]. Therefore, the torpor optimization hypothesis predicts that YOY should use their energy reserves more thriftily than adults, as in juvenile *S. richardsonii* which enter hibernation with lower fat masses than adults, but undergo a smaller decrease in fat content throughout hibernation [32]. On the other hand, YOY may be less skilled at budgeting energy during winter, which could lead to a faster decline in their reserves. Despite these potential differences in hibernation strategies and/or abilities of YOY, they have not been distinguished from adults in past studies of fat depletion by hibernating bats [36], [51], [52].

Body condition is a measure of energy reserves relative to a structural element of the body [53]. As both resource availability and energetic demands shift seasonally, body condition fluctuates. For fat-storing hibernators during winter this fluctuation is simple: with little or no opportunity for energy acquisition, body condition declines until spring. The thrifty female hypothesis predicts that, throughout the hibernation period, females will undergo a smaller decline in body condition than males. To test this prediction, we assessed the body condition of adult male and female little brown bats just before hibernation during fall swarming, during early hibernation, and just before emergence in the spring. We also assessed changes in body condition of YOY to determine if they are more conservative in their use of fat reserves than adults.

## Methods

This study was conducted north of Grand Rapids (population ∼300) in central Manitoba, Canada (53°30′N; 99°24′W; not to be confused with Grand Rapids, Michigan, USA). The region is composed of mature boreal forest with extensive limestone karst topography. Over 50 caves have been surveyed in the area, including five confirmed little brown bat hibernacula: Dale's Cave, Firecamp Cave, The Abyss and (in the Walter Cook Caves Park Reserve) Microwave Cave and Iguana Crypt. The greatest straight-line distance between any of these caves (Abyss and Iguana) is 31 km. Spring surveys indicate that populations of bats in each cave range between about 50 and 500 individuals each winter, although most sites housed several hundred bats (J. Dubois and C.K.R. Willis, unpublished data). The hibernation period in this region lasts approximately eight months from mid-September until mid-May. Temperature data loggers (iButtons, Maxim Integrated Products, Dallas, Texas, USA) wrapped in insulating foam to attenuate ultrasonic noise [54] were used to record ambient temperature (T_a_) every two hours in Dale's, Firecamp and Microwave. As much as possible, we placed dataloggers in areas known to be used by bats to ensure recording of microclimates that bats actually used (particularly in domes or cracks). Microclimates were similar between caves, which varied between average minima of 0.8±1.8°C and average maxima of 8.1±1.2°C during hibernation (Figure S1).

Bats were captured with harp traps (G5, Bat Conservation and Management, Carlisle PA, USA) biweekly during the swarming period (15 Aug–1 Oct 2009) near the entrances to Dale's, Microwave, Firecamp and Abyss. We used data collected during 15–19 Sept 2009 as the sample for pre-hibernation BCI of bats because by this time at least a few bats had already entered hibernation while some remained active, which means we sampled as close to the start of hibernation as possible. During early hibernation, torpid bats were captured by hand from Microwave (28 Nov 2009), Firecamp (28 Nov 2009) and Dale's (29 Nov 2009). Near the end of hibernation, bats were captured from Microwave (27 April 2010), Firecamp (27 April 2010) and Iguana Crypt (28 April 2010). We did not re-enter Dale's in late hibernation 2010 to minimize disturbance to these bats, which were also part of another study. Instead we measured bats from Iguana Crypt, a similar cave in the region. Bats were sexed, weighed to the nearest 0.01 g (Durascale-100, MyWeigh, ON, Canada) and their forearm length was measured to the nearest 0.05 mm. We classified individuals as YOY or adults based on ossification and shape of wing joints [50], tooth wear [55], and evidence of previous reproduction [56]. We were readily able to age bats throughout hibernation which suggests that the short active season in this region may not permit YOY to fully mature before their first winter. At the time age was determined we were blind to the body condition of individuals. All bats were outfitted with a numbered, lipped aluminum forearm band (2.9 mm, Porzana Ltd., East Sussez, UK) prior to their release at the site of capture. All methods were approved by the University of Winnipeg Animal Care Committee and conducted under Manitoba Conservation Wildlife Scientific Permit WB06122.

We defined body condition index as mass divided by forearm length which has been validated in temperate bats [57], [58]. Variation in mass can be largely attributed to pre-hibernation fattening and subsequent use of these reserves to fuel hibernation because during hibernation little brown bats do not feed [59] and muscle mass does not decline appreciably [60].

To assess differences in BCI between age/sex classes for each sampling period, we used ANOVA with Tukey's test for pair-wise comparisons, or non-parametric Kruskal-Wallis tests as appropriate. Data from all swarming sites were pooled because bats were likely members of the same population, sample sizes at individual capture sites were low and capture site had no effect on BCI during swarming (ANOVA, F_3,59_ p = 0.14). Data from all caves were pooled as capture site had no effect on BCI during early hibernation (F_2,113_ = 0.44, p = 0.65) or during late hibernation (F_2,97_ = 1.8, p = 0.18). We calculated effect size (i.e., Cohen's d) to compare the magnitude of differences between adult males and females during each sampling period. This provided a quantitative indication of the relative difference between sex classes at different times during hibernation [61]. We used SYSTAT 11 (Systat Software Inc., Point Richmond, CA, USA) for all significance tests based on an alpha level of p<0.05. Effect sizes were calculated using Watkins' [62] Effect Size Calculator.

## Results

We captured a total of 432 little brown bats (72 adult females, 137 adult males, 100 YOY females and 123 YOY males). Of these, 200 were captured swarming around the four cave sites (22 adult females, 44 adult males, 65 YOY females, and 69 YOY males), 125 were captured during early hibernation from Dale's (n = 22), Firecamp (n = 51), and Microwave (n = 52), and 107 were captured during late hibernation from Iguana Crypt (n = 28), Firecamp (n = 34), and Microwave (n = 45). Eight bats captured during swarming were recaptured during early hibernation, 20 bats captured during early hibernation were recaptured during late hibernation and three bats captured during swarming were recaptured during late hibernation. Results reported below are based on analyses which included this small proportion of recaptured bats. However, when we re-analysed the data excluding recaptures, there was no change in any of our conclusions. Proportionally more YOY were captured during swarming than during early or late hibernation but sex ratios were virtually identical between early and late hibernation (Figure S2). Bats weighed 12.06±1.11 g (mean ± SD) just prior to the beginning of hibernation (i.e., at immergence into hibernation), 10.08±0.95 g during early hibernation and 8.47±1.01 g during late hibernation.

### Swarming

**Figure 1:**
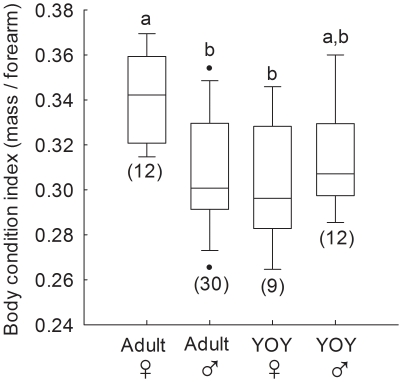
Body condition indices of bats during fall swarming. Bats were captured from 15–19 Sept 2009 at the entrances of Abyss, Dale's, Firecamp and Microwave caves. Numbers in brackets indicate sample size and boxes sharing the same letter are not significantly different from each other. Boxes depict the 25^th^ and 75^th^ percentiles, lines within boxes mark the median, whiskers represent 95^th^ and the 5^th^ percentiles and dots indicate outliers.

During the swarming period, near the time of immergence, there was a significant effect of age/sex class on BCI (ANOVA, F_3,59_ = 5.6, p = 0.002; Figure 1). Values for active adult females were significantly greater than those of adult males (Tukey's test, p = 0.002) and YOY females (p = 0.005), and the difference between adult females and YOY males approached significance (p = 0.051). There were no significant differences between adult males, YOY males or YOY females. Effect size for the difference in BCI between adult males and adult females equated to a 40.7% overlap between the two frequency distributions (Cohen's d = 1.1).

### Early Hibernation

**Figure 2:**
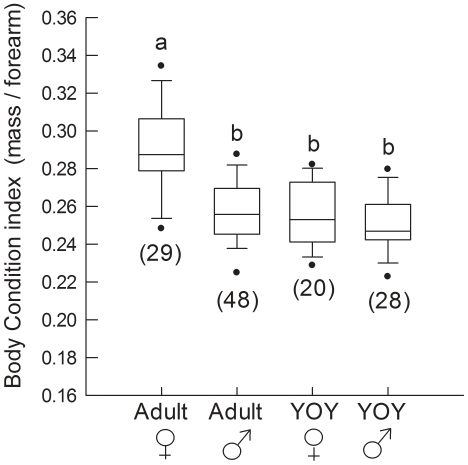
Body condition indices of bats during early hibernation. Bats were captured on 28, 29 Nov 2009 in Dale's, Firecamp and Microwave caves. Numbers in brackets indicate sample size and boxes sharing the same letter are not significantly different from each other. Boxes depict the 25^th^ and 75^th^ percentiles, lines within boxes mark the median, whiskers represent 95^th^ and the 5^th^ percentiles and dots indicate outliers.

During early hibernation the effect of age/sex class on BCI was highly significant (F_3,121_ = 27.1, p<0.001; Figure 2). Adult females were in significantly better body condition than adult males (p<0.001), YOY females (p<0.001) or YOY males (p<0.001). There were no significant differences between adult males, YOY males or YOY females. This pattern was consistent across cave sites, and a significant effect of age/sex class on BCI was observed in Dale's (F_3,18_ = 9.5, p = 0.001), Firecamp (F_3,47_ = 15.1, p<0.001) and Microwave (F_3,48_ = 6.6, p = 0.001). In all caves, adult females were in significantly better body condition than adult males (p≤0.001) and YOY males (p≤0.008). Adult females were in better condition than YOY females in Dale's and Firecamp (p≤0.003), but not Microwave (p = 0.089). In all caves, there were no significant differences between adult males, YOY males or YOY females. The effect size for the difference in BCI between adult males and adult females equated to a 27.3% overlap in the two frequency distributions (Cohen's d = 1.6), which was a larger effect than that during swarming. The percent decline in BCI was greatest between swarming and early hibernation for all age/sex classes. During this interval, percent decline in BCI was similar for adult females, adult males and YOY females, while BCI of YOY males declined more rapidly (Table 1).

**Table 1:** Average percent decline in little brown bat body condition index (BCI; mass/forearm length) and rate of mass loss between swarming (15–19 Sept 2009), early hibernation (28, 29 Nov 2009) and late hibernation (27, 28 April 2010).

		Adult female	Adult male	YOY female	YOY male	All females	All males	All age/sex classes
Swarming – Early Hibernation	% Autumn BCI	15.20%	16.50%	15.50%	20.10%	12.40%	17.20%	16.20%
(71 days)	mg/day	26.8	28.3	23.2	35.1	24.8	30.6	27
Early – Late Hibernation	% Autumn BCI	9.40%	14.20%	13.20%	12.70%	12.10%	14.00%	13.30%
(140 days)	mg/day	8.9	11.4	12	10.8	11	11	11
Swarming – Late Hibernation	% Autumn BCI	24.60%	30.70%	28.70%	32.80%	24.40%	31.20%	29.50%
(211 days)	mg/day	14.9	17.1	15.8	19	16	18	17
	g/240 days*	3.58	4.1	3.78	4.56	3.72	4.27	4.08

*Duration of hibernation season in Ontario (Fenton, 1970). This value has been used by other several other studies of winter energy budgets, although it may be shorter than the duration of hibernation for individuals from our more northerly study site.

### Late Hibernation

**Figure 3:**
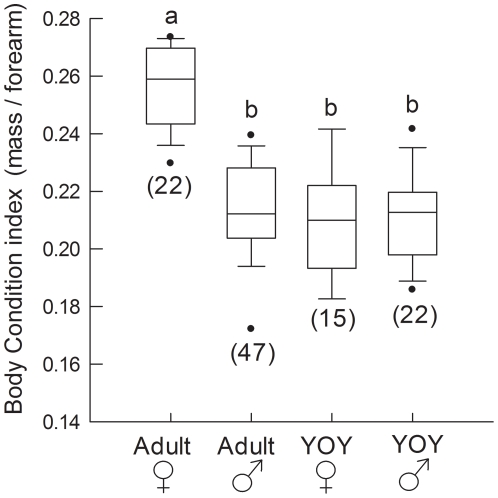
Body condition indices of bats during late hibernation. Bats were captured on 27, 28 Apr 2009 in Iguana, Firecamp and Microwave caves. Numbers in brackets indicate sample size and boxes sharing the same letter are not significantly different from each other. Boxes depict the 25^th^ and 75^th^ percentiles, lines within boxes mark the median, whiskers represent 95^th^ and the 5^th^ percentiles and dots indicate outliers.

During late hibernation the effect of age/sex class on BCI was highly significant (F_3,105_ = 38.3, p<0.001; Figure 3). Adult females were in significantly better body condition than adult males (p<0.001), YOY females (p<0.001) or YOY males (p<0.001). There were no significant differences between adult males, YOY males or YOY females. This pattern was consistent across cave sites, and a significant effect of age/sex class on BCI was observed in Iguana (F_3,24_ = 8.3, p = 0.001), Firecamp (F_3,30_ = 8.5, p<0.001) and Microwave (F_3,41_ = 18.5, p<0.001). In all caves adult females were in significantly better body condition than adult males (p≤0.009), YOY females (p≤0.011), and YOY males (p≤0.003) and there were no significant differences between adult males, YOY males or YOY females. Effect size for the difference in BCI between adult males and females was greatest during this sampling period (Cohen's d = 2.4) and represented only a 12.5% overlap between the two frequency distributions.

**Figure 4:**
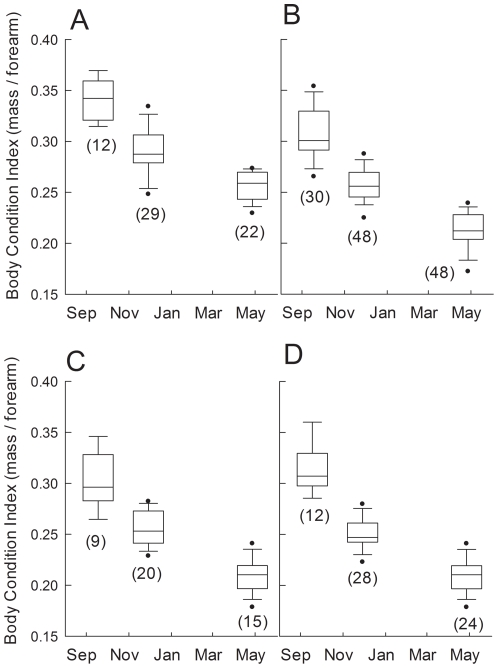
Body condition indices of bats throughout the hibernation period. A) adult females, B) adult males, C) young-of-the-year females and D) young-of-the-year males. Data from all caves are pooled and numbers in brackets represent pooled sample sizes for each sampling period. Boxes depict the 25^th^ and 75^th^ percentiles, lines within boxes mark the median, whiskers represent 95^th^ and the 5^th^ percentiles and dots indicate outliers.

Between early hibernation and late hibernation the decline in BCI of adult males was the greatest, followed by that of YOY females, YOY males and adult females (Table 1). By late hibernation adult females had experienced the lowest percentage decline in BCI, followed by YOY females, adult males and YOY males (Table 1). Moreover, the magnitude of difference in BCI between adult females and adult males, as represented by effect size, progressively increased throughout hibernation from a relatively small effect during fall swarming (Cohen's d = 1.1), to an intermediate effect size in early hibernation (d = 1.6), to a large effect by spring (d = 2.4; Figure 4).

## Discussion

Our findings support the prediction of the thrifty female hypothesis that female little brown bats should exhibit a slower decline in body mass during winter than males. Not only was the decline in BCI for adult females (24.8%) less than that of adult males (30.7%), the magnitude of difference in adult male and female BCI steadily increased throughout hibernation. Some of this difference could reflect the allometry of metabolic rate which predicts that smaller bats (i.e., males) should decline in body condition faster than larger ones (i.e., females) simply because of higher mass-specific metabolic rates [63]. However, the effect of body mass on mass-specific metabolic rate is negligible during deep torpor [64] and male bats still exhibited a greater rate of decline in total fat reserves (i.e., a greater whole-animal metabolic rate; 112 mg fat/week vs. 98 mg fat/week for females). Therefore, differences in rates of decline in BCI were greater than predicted based on body size alone (Table 1). Our data also suggest that this difference is more strongly influenced by avoidance of physiological/ecological costs of torpor rather than by increased male activity due to mating. During early hibernation, when mating frequency is highest, the average decline in adult male BCI was only 106% that of adult females (Table 1) [39]. On the other hand, during late hibernation, when hibernaculum temperatures are lower (Figure S1) and, presumably the physiological/ecological costs of torpor would be greater, the decline in BCI for males was nearly 130% that of females (Table 1). Thus, males lost less fat, relative to females, during early hibernation. If the difference in rates of decline was primarily the result of mating activity by males, the opposite pattern should occur. Therefore, our findings are consistent with the hypothesis that males spent more energy to avoid costs of torpor.

The torpor optimization hypothesis generates conflicting predictions for adult female little brown bats. Females enter hibernation with larger fat reserves than males and are, therefore, predicted to use their excess energy to reduce use of torpor. However, they need additional energy to support spring reproduction, which means they should conserve energy by increasing use of torpor. Our findings support Humphries *et al.* [28] prediction that individuals of the sex which depends on spring fat reserves to reproduce should rely more heavily on torpor to help hoard resources for reproduction. Conserving resources for spring reproduction appears to take precedence over mitigating potential costs of torpor. Echidnas are known to use a similar approach, employing facultative hibernation if they are in good body condition to maintain reserves until the reproductive season, whereas those with insufficient reserves continue to forage [65]. The thrifty female strategy may also apply to other species of temperate, hibernating bats. For example, female *E. fuscus* and *Perimyotis subflavus* both decline in mass at a slower rate than males during hibernation [51], [52].

In addition to being thrifty in their fat expenditure during winter, accumulating more fat prior to hibernation appears to be a second way that females increase their chances of successful reproduction. Adult females in our study entered hibernation in better condition than adult males or YOY, which is consistent with past studies of little brown bats based on body fat indices or body mass [48], [49], [50] and that of other temperate, hibernating bat species, including *Eptesicus fuscus*, *Perimyotis subflavis*, *M. yumanensis* and *M. thysanodes* [48], [51], [52]. In several species of vespertilionid bats, including little brown bats, females are structurally larger and have proportionately greater wing areas than males [66], [67]. This sexual dimorphism may be driven by demands of flying with a large fetus (∼25% of maternal weight [66]) and/or the pressure to reduce heat loss and avoid torpor during summer while rearing young [67]. However, a larger body size could also allow females to store and carry proportionally more body fat than males prior to hibernation without substantially compromising flight ability. On the other hand, males may enter hibernation in worse condition than females simply because mating prior to hibernation is more costly to males than females [37], [49].

Females that emerge from hibernation with greater energy reserves likely have improved reproductive success. For example, in female Columbian ground squirrels (*Spermophilus columbianus*), spring emergence weight is positively correlated with the number of young that survive their first winter [68]. Female bats with larger remaining fat reserves may be able to emerge from hibernation earlier in the spring because they have energy available to endure periods of inclement weather and low insect availability [69]. This could allow for earlier parturition, which would be beneficial because bats born earlier have a greater chance of surviving their first winter and breeding their first year [11], [70].

We found no evidence to support the hypothesis that YOY hibernate more conservatively than adults, despite smaller pre-hibernation energy reserves. Both male and female YOY declined in BCI more rapidly than their adult counterparts. It is possible that YOY are inexperienced and require time to learn behaviours important for conserving fat (e.g., microclimate selection, choice of huddling partners). In other species behaviours associated with hibernation must be learned. For example, the hibernation burrows of juvenile Columbian ground squirrels are often poorly constructed and too shallow relative to those of adults [71]. Moreover, although body size did not appear to influence relative rates of fat loss for adult males and females, if YOY are smaller than adults, with greater surface-area to volume ratio, their inexperience could exacerbate the effects of a smaller body size (i.e., higher rates of heat loss and greater energetic costs of euthermia during periodic arousals). Adult alpine marmots (*Marmota marmota*) reduce the energetic cost of euthermic periods for YOY by huddling with them in family groups and arousing synchronously [72]. It is not known if bats maintain similar social connections with relatives during hibernation, but if clustering increases inclusive fitness, relatedness could help explain bats' choices of huddling partners within hibernacula.

On average, the over-winter decline in fat reserves for the little brown bats we studied in Manitoba, Canada (29.5% or 119 mg/week; Table 1) was nearly identical to the 29.3% mass loss predicted by Thomas, Dorais & Bergeron [73], but greater than the 25% over-winter decline in body mass (or 91 mg/week) observed between October and April in an Ontario population of this same species [36]. Fenton [36] did not detect a difference in rates of fat loss between males and females, but he did not differentiate YOY from adults, which may have obscured sex differences. To test this possibility we re-analyzed our data without differentiating YOY from adults (Table 1). Although we still found significant differences between sexes, failing to differentiate bats by age decreased effect sizes and partially masked the difference between males and females. Therefore, we recommend that future studies of hibernation energetics in bats aim to differentiate YOY from adults.

For all age/sex classes the most rapid weight loss occurred between swarming and early hibernation (Table 1), which could reflect two factors. First, loss of mass during early hibernation may actually be greater than during late hibernation because average T_a_ in the hibernaculum is higher early in the winter and above “optimal” for torpor expression (Figure S1). Ideal T_a_ for hibernating little brown bats has usually been considered to be about 2°C [74], [75], although Boyles & McKechnie [76] recently presented evidence that temperatures several degrees higher may be optimal if hibernaculum microclimate fluctuates. Warmer T_a_ reduces torpor expression [77], [78] and, therefore, energetic savings. This could make early hibernation more energetically costly than late hibernation, but more favorable in terms of physiological/ecological costs of torpor. Second, despite our efforts to capture bats early in the night before feeding, and hold them until they had defecated, swarming bats likely had gut contents which may have caused us to overestimate body condition. Differential mortality among age/sex classes could also explain the pattern we observed. However, mortality was low throughout the study (n = 3), despite the fact that caves were small and dead bats were easily found. More important, we observed virtually identical age/sex class ratios in early and late hibernation (Figure S2), which means the same proportion of bats from each age/sex class were still alive in spring. This suggests that the pattern we observed, of a falling rate of decline in BCI later in the winter, especially for adult females, accurately reflects how bats expend their energy reserves during winter.

Given links between WNS and hibernation energy balance [6], [10], our results have implications for the survival and reproduction of bats suffering from this new disease. No data are available to determine if WNS affects age/sex classes differentially, but if afflicted individuals adjust hibernation patterns in reference to their “normal” rates of fat depletion, then our results predict that adult females will be most likely to survive the disruption of energy balance caused by WNS because they enter hibernation with proportionally greater energy reserves and use these reserves more conservatively. Affected females could therefore use resources allocated for reproduction to fuel the additional time out of torpor which appears to be caused by WNS [9] and may be more likely to survive until the end of hibernation. However, females that emerge with BCI similar to males will likely have lower levels of reproductive success and may even forgo reproduction. Frick *et al.* [11] observed a decline in the proportion of reproductive females at a maternity colony in New Hampshire co-incident with the spread of WNS. The females they observed may have survived hibernation by expending fat normally reserved for reproduction. More data are clearly needed on overwinter survival of WNS-affected male bats but differential mortality/reduced reproduction could have important consequences for populations and for models which aim to predict impacts of WNS for populations.

Our results may also have implications for how WNS is spreading throughout North America. Male survivors of WNS may be more likely to be vectors for the fungus than females, as males likely use colder summer roosts than females, where fungal conidia may be more likely to persist [7]. In addition, although data are scarce, anecdotal evidence suggests males may be more likely to make large-scale migrations between mating swarms/hibernacula, at least in little brown bats [36], [79]. If males are less likely to survive but more likely to spread the disease then the differential mortality predicted by our results could mean that the rate of spread is actually slower than a “worst-case scenario” because the most likely summer vectors for the fungus (i.e., males) may be the first to be killed. More work is needed to test these hypotheses and it is also critical to determine the persistence of fungal material on bats or in summer roosts of both males and females to better understand how WNS is spreading in the wild.

Our results suggest that strong selection pressure acts on female little brown bats to ensure energy availability for spring reproduction because females enter hibernation with larger reserves and use these reserves more conservatively. This pattern could also increase the likelihood that females will survive disruptions to hibernation such as WNS, although WNS will likely have consequences for reproduction, and is clearly still causing mortality of enormous numbers of females. Young-of-the-year used their hibernation energy reserves more rapidly than adults, but we observed no evidence of higher levels of winter mortality. This suggests that, at least in some years at some sites, juvenile mortality is not higher than that of adults although this is commonly cited in the literature [50], [80], [81]. Our study emphasizes the need to consider how age and sex influence variation in energy expenditure in free-ranging bats and the potential for differential mortality to influence population trends and responses to disturbances like WNS.

## Supporting Information

Figure S1 **Monthly average ambient temperature in hibernacula.** Mean ± standard deviation in A) Dale's cave B) Firecamp cave C) Iguana crypt and D) Microwave cave. Closed circles denote the winter of 2008/2009 (beginning on 17 September) and open circles denote the winter of 2009/2010. Diamonds indicate data from Bilecki [82].
(TIF)

Figure S2 **Proportion of age/sex classes captured.** Bats were captured during fall swarming (15 Aug–1 Oct 2009), early hibernation (28, 29 Nov 2009), and late hibernation (27, 28 April 2010).
(TIFF)
