# Supplementary figures and images for: Changes in Body Condition of Hibernating Bats Support the Thrifty Female Hypothesis and Predict Consequences for Populations with White-Nose Syndrome
Source: PLoS One. 2011 Jun 22;6(6):e21061. doi: 10.1371/journal.pone.0021061 (PMC3120823; doi:10.1371/journal.pone.0021061)

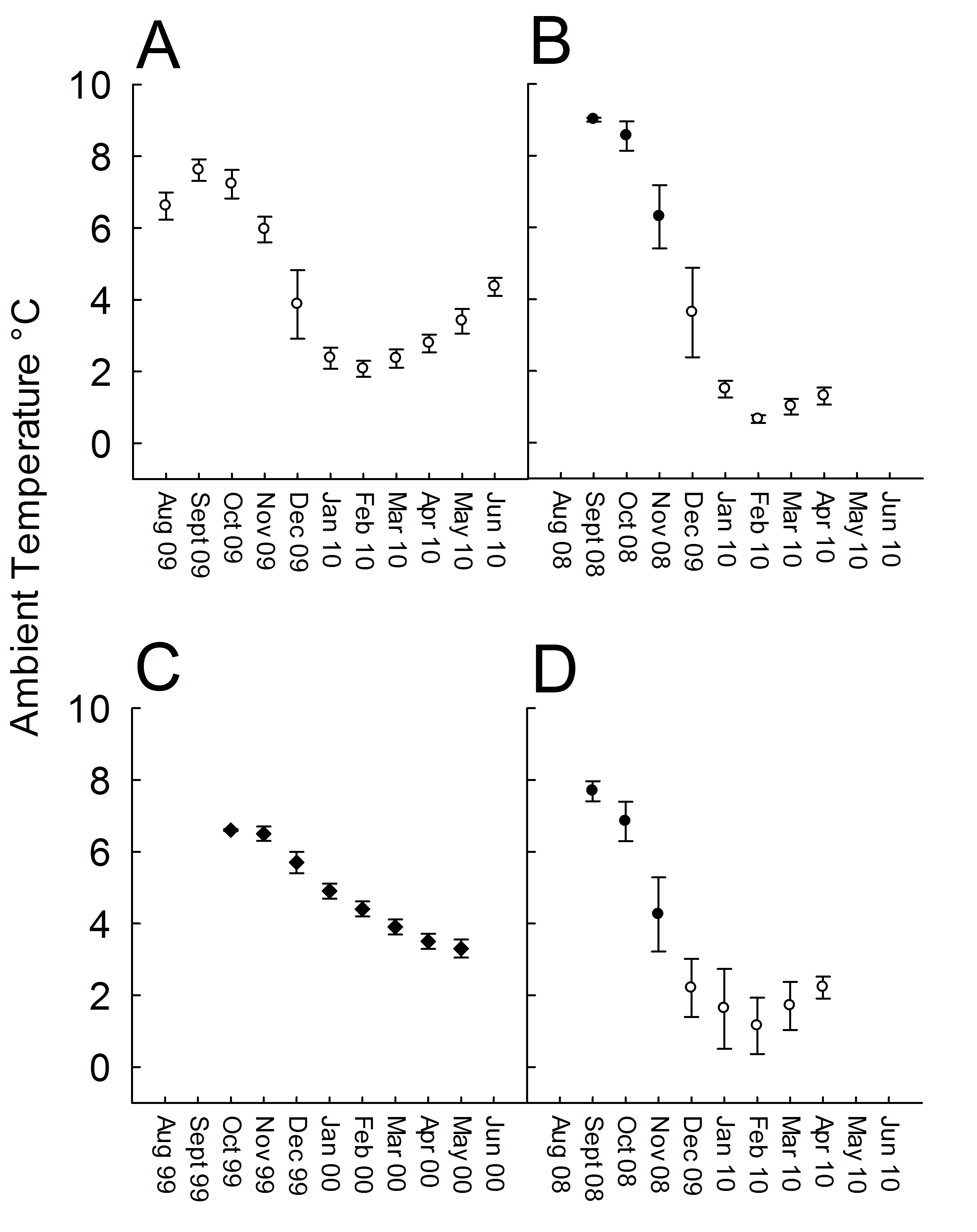

Supplement: Figure S1 — Monthly average ambient temperature in hibernacula. Mean ± standard deviation in A) Dale's cave B) Firecamp cave C) Iguana crypt and D) Microwave cave. Closed circles denote the winter of 2008/2009 (beginning on 17 September) and open circles denote the winter of 2009/2010. Diamonds indicate data from Bilecki [82]. (TIF) [file pone.0021061.s001.tif]

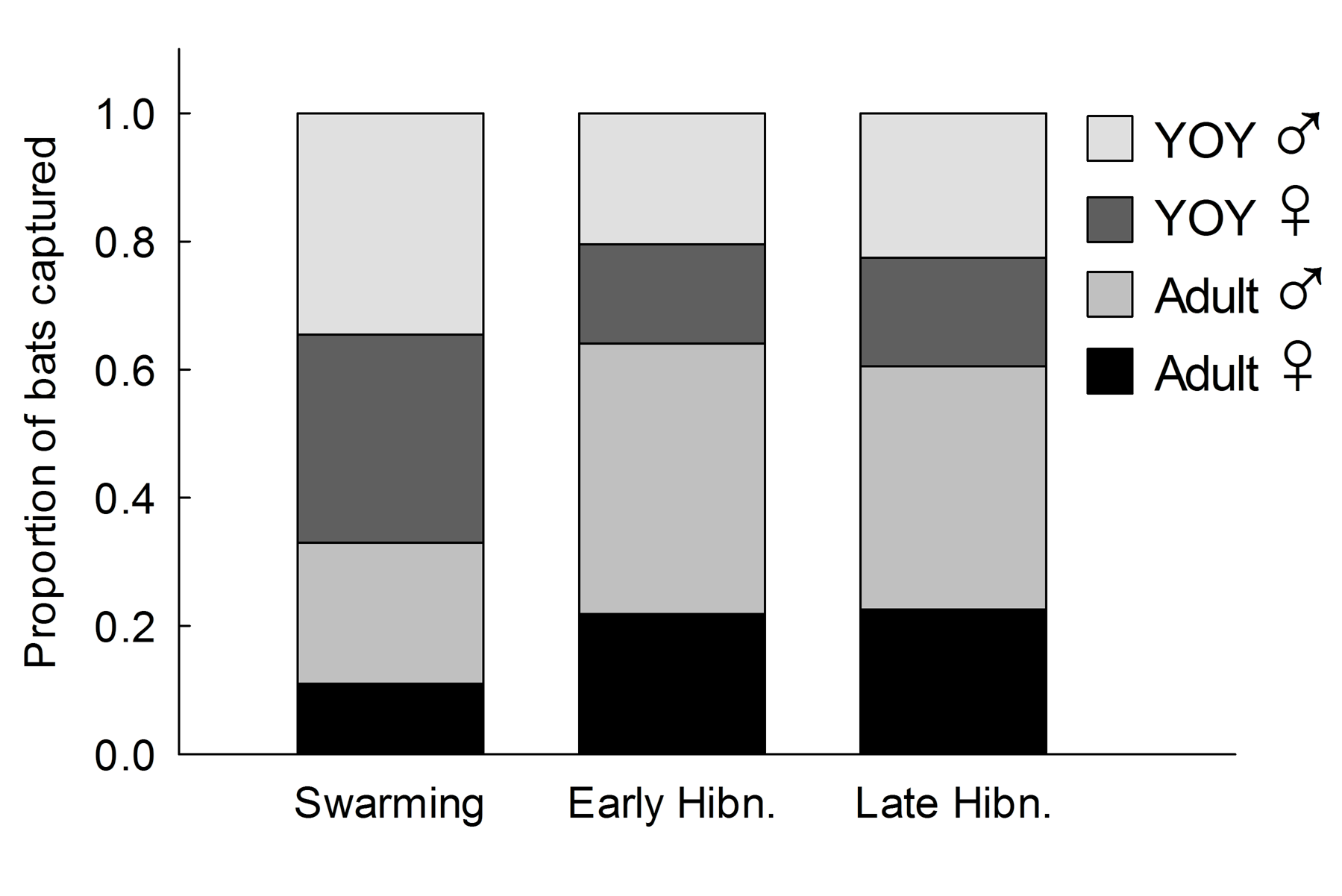

Supplement: Figure S2 — Proportion of age/sex classes captured. Bats were captured during fall swarming (15 Aug–1 Oct 2009), early hibernation (28, 29 Nov 2009), and late hibernation (27, 28 April 2010). (TIFF) [file pone.0021061.s002.tiff]
